# Editorial: Molecular and cellular mechanisms of sensory functions in insect models

**DOI:** 10.3389/fnmol.2022.1064452

**Published:** 2022-10-21

**Authors:** Takaaki Sokabe, Youngseok Lee, Jia Huang

**Affiliations:** ^1^Division of Cell Signaling, National Institute for Physiological Sciences, and Thermal Biology Group, Exploratory Research Center on Life and Living Systems (ExCELLS), National Institutes of Natural Sciences, Okazaki, Japan; ^2^Department of Bio & Fermentation Convergence Technology, Kookmin University, Seoul, South Korea; ^3^Institute of Insect Sciences, Zhejiang University, Hangzhou, China

**Keywords:** sensory function, *Drosophila melanogaster*, receptor, behavior, ion channel, sensory neuron, crop pest, disease vector

Sensory functions are an initial step in communicating with our environment. All biological systems need to monitor fluctuation of the surrounding physical/chemical parameters to respond to adjust their inner states and adapt to new circumstances. Recent advances in Sensory Biology have identified a diverse set of molecules involved in the sensory functions. There exists, however, a number of uncertainties regarding how the environmental signals are received, converted, and transmitted by those sensory molecules, which consequently affect behavioral outputs. In the last two decades, fruit flies (*Drosophila melanogaster*) have emerged as a powerful model in Sensory Biology studies. This tiny organism is quite sensitive to changes in their environment and immediately react with simple and robust behaviors, relying on their simple neural circuits. In addition to fruit flies, the importance of insect studies has been growing particularly in insect pests exemplified by mosquitoes and brown planthoppers, a threat for public health and crops, respectively. Unraveling these complex sensory processes in insect models, therefore, is beneficial for basic neuroscientific research and in the development of new strategies of pest management.

In this Research Topic, a variety of articles were provided by dedicated contributors, representing the diversity and the complexity of Sensory Biology studies in flies and insect pests.

The transient receptor potential (TRP) channel family is involved in almost every sensory modality. There are about 30 members of TRP channels in insect genomes, and most of them are expressed in sensory neurons thereby controlling behaviors related to vision, smell, taste, hearing, thermosensation, nociception, proprioception and others. However, it is less understood whether TRP channels are involved in reproduction processes such as an egg laying behavior. Zhang et al. found that TRPL, a member of the TRPC subfamily, regulated egg laying both in fruit flies and brown planthoppers, a rice pest. Knocking down the planthopper *trpl* led to a significant reduction in egg laying. The *Drosophila trpl* reporter line labeled sensory neurons in female oviduct and egg-laying behavior was impaired in the *trpl* mutants. More importantly, such defects were rescued either by fly or planthopper *trpl* transcript, indicating an evolutionary conserved role of this ion channel.

Class III (CIII) neurons function as a multimodal sensor in *Drosophila melanogaster*, and intracellular Ca^2+^ level plays key roles in modality-specific neural and behavioral responses. Patel et al. identified that metabotropic GABA_B_-R2 receptor and Ca^2+^ induced Ca^2+^ release (CICR) *via* inositol trisphosphate receptor (IP_3_R) were specifically responsible for noxious cold responses. In contrast, in the same neurons, CICR *via* the ryanodine receptor (RyR) was generally required for cold nociception and innocuous touch response. Both the GABA_B_-R2 and RyR contributed to cooling-induced activity of CIII neurons and firing patterns. The authors also confirmed that Pkd2, one of the TRP channels in CIII neurons, was involved both in cold nociception and touch sensation, whereas IP_3_R was responsible only for cold nociception. These results reveal the multiple yet partially overlapping sets of molecules that could be important for modality specific intracellular Ca^2+^ levels and behavioral outputs.

*Aedes aegypti* (*Aa*), an infamous disease vector for yellow fever, utilizes auditory function to discriminate wing beat frequency and it is particularly important for males to target conspecific females for courtship, which is called phonotaxis. Xu et al. showed that serotonin receptors 5-HT_7A_ and 5-HT_7B_ were abundantly expressed in the Johnston's organ (JO), an auditory unit in the head, and their pharmacological manipulation altered the properties of JOs in *Aa* males: stimulating with serotonin injection induced shifts in male ear tuning to higher frequencies, while inhibition of serotonin synthesis led to a reduction in tuning frequencies. The authors also demonstrated an interference of *Aa* male phonotactic behaviors by inhibiting serotonin synthesis, which resulted in reduction of responsiveness to sounds and the range of frequencies that the males responded. The serotonergic system in *Aa* mosquitoes could be a new target for vector control.

Taste sensation is critical for final decision making upon nutrient uptake and diverse sets of Gustatory receptors (Grs) play major roles in various regions of the insect body. Kohatsu et al. dissected the roles of Gr5a-positive and Gr61a-positive gustatory receptor neurons (Gr5a+ and Gr61a+ GRNs, respectively) and found that the former predominantly controlled the preference of trehalose over other sugars and meal size, while the latter affected only sugar preference. They labeled the neurons with Gr5a- and Gr61a-Gal4 drivers in tarsal segments and found two distinct populations: Gr5a-/Gr61a+ and Gr5a+/Gr61a+ GRNs. These GRNs projected separately to the CNS to form alternative neural circuits, and the authors propose that the two types of GRNs/sensory pathways may function in different gustatory responses.

Among the interesting contributions, Schoberleitner et al. proposed a model fly which may be associated with age-related decline processes in animals. The ATP-dependent chromatin remodeling factor chromodomain-helicase-DNA binding protein 1 (CHD1) had a crucial role in the incorporation of the histone variant H3.3 in the fly brain. The *Chd1* mutant flies resulted in widespread upregulation of transcription including the control of hunger and satiety-related genes. The mutants had impairments in nutrient metabolism and food intake. Furthermore, *Chd1* mutant flies showed impairments in olfactory and gustatory perception, but not visual perception. The mutants also had defects in climbing and exploratory walking. Most phenotypes were rescued by expression of the *Chd1* cDNA using pan-neuronal GAL4. However, the olfactory defects were suggested by indirect non-neuronal regulation.

All the articles above advance our understandings and provide insight into the molecular machineries of sensory functions. This trend has been expanding in the last two decades, and the Sensory Biology field is indeed widely acknowledged in the scientific communities now, as the Nobel Prize in Physiology or Medicine in 2021 was awarded for “the discoveries of receptors for temperature and touch”. Future work will further prove the advantages of insect models in Sensory Biology that benefits basic research as well as applications for insect pest management.

## Author contributions

All authors listed have made a substantial, direct, and intellectual contribution to the work and approved it for publication.

## Conflict of interest

The authors declare that the research was conducted in the absence of any commercial or financial relationships that could be construed as a potential conflict of interest.

## Publisher's note

All claims expressed in this article are solely those of the authors and do not necessarily represent those of their affiliated organizations, or those of the publisher, the editors and the reviewers. Any product that may be evaluated in this article, or claim that may be made by its manufacturer, is not guaranteed or endorsed by the publisher.

